# Mental Health Status of Parental Caregivers of Special Needs Children in Puducherry

**DOI:** 10.5041/RMMJ.10551

**Published:** 2025-07-31

**Authors:** Philip Felix Priya, Ananda Balayogi Bhavanani, Meena Ramanathan, Karthick Subramanium, Sukanto Sarkar, Anandraj Lokeshmaran

**Affiliations:** 1 School of Yoga Therapy, Institute of Salutogenesis and Complementary Medicine, Sri Balaji Vidyapeeth (Deemed-to-be-University), Puducherry, India; 2 Institute of Salutogenesis and Complementary Medicine, Sri Balaji Vidyapeeth (Deemed-to-be-University), Puducherry, India; 3 Department of Psychiatry, Mahatma Gandhi Medical College & Research Institute, Sri Balaji Vidyapeeth (Deemed-to-be-University), Puducherry, India; 4 Department of Psychiatry, All India Institute of Medical Sciences, Kalyani, West Bengal; 5 Department of Community Medicine, Mahatma Gandhi Medical College & Research Institute, Sri Balaji Vidyapeeth (Deemed-to-be-University), Puducherry, India

**Keywords:** Anxiety, caregiver burden, caregivers, depression, quality of life, special needs children

## Abstract

**Background:**

Parental caregivers of children with special needs manage their child’s daily tasks, taking on responsibilities such as making health and financial decisions, assisting with routine activities, and ensuring their safety from self-harm. The level of a child’s disability determines the amount of time and effort a caregiver must invest, with higher disability levels meaning greater dependency and thus requiring greater support. While rewarding, caregiving may also be highly demanding. The parental caregiver’s physical and mental health can decline due to stress, potentially leading to anxiety and depression, and may worsen pre-existing conditions. This research aimed to provide insights into the psychological well-being of parental caregivers, shedding light on their challenges and needs for better support and intervention.

**Methods:**

This study examined the psychological health of parental caregivers of special needs children at a special education school in Puducherry, India. Following an orientation program, 66 parental caregivers volunteered and provided informed consent to participate. The mean age of the parents was 38.4 years (±6.6). Demographic details were collected, and psychological parameters were assessed using standardized scales: Zarit Burden Interview–Caregiver Burden Scale (ZBI-CBS), Depression Anxiety Stress Scale (DASS-21), Pittsburgh Sleep Quality Index (PSQI), World Health Organization Quality of Life–brief form (WHOQOL-BREF).

**Results:**

The study findings revealed that most parents experienced caregiver burden and poor sleep quality, consistent with previous studies. Specifically, 93.9% of parents had poor sleep, while 84.8% reported caregiver burden. Additionally, 89.4% of the parents experienced depression, 89.4% have anxiety, and 86.4% have stress. Quality of life was low across all domains.

**Conclusion:**

The stress of managing their child’s needs can negatively impact parental caregivers’ physical and psychological health. Providing counseling and promoting healthy lifestyle choices can significantly enhance caregivers’ overall well-being.

## INTRODUCTION

Parental caregivers of children with special needs handle their child’s daily tasks, with involvement varying based on the child’s dependency. This includes assistance with eating, bathing, medication, activity planning, transportation, safety, and making financial and health decisions, among other responsibilities.

Caregiving can be rewarding, providing a sense of duty fulfilment, but it is also very challenging. Over time, parental caregivers often experience increased stress, which can deteriorate their psychological well-being and physical health.^1^

According to the 2011 Census of India, 2.2% of the population had a physical or mental disability and 1.6% of Tamil Nadu’s population had disabilities.^2^ According to the Child Rights Information Network (CRIN), in India, children constitute 35.29% of the total disabled population, with the highest proportion of disabilities found in the 10 to 19 age group.^3^ Caring for children with disabilities can be demanding and significantly impacts their families.^4^

The aim of the study was to explore the caregiving burden, stress levels, sleep quality, and quality of life of parental caregivers.

## MATERIALS AND METHODS

This study focused on parental caregivers of children with special needs under the age of 18, residing within Pondicherry and Tamil Nadu and enrolled in a special education institution in Pondicherry. Ethical clearance was obtained from the Institutional Human Ethics Committee (Ph.D. PROJECT/C4/ 2019/D06) of the Mahatma Gandhi Medical College & Research Institute.

### Study Participants and Definitions

Study participants were selected from among the parents of special needs children studying at a special education school. An introductory orientation session helped potential participants understand the study objectives and outlined participant roles, thereby ensuring a uniform and comprehensive understanding of the study and promoting effective cooperation. After attending the session, 82 parents completed an application to participate in the study; of these, 66 parents were selected in accordance with predetermined inclusion and exclusion criteria. Informed consent was obtained from all participants. Eligible participants included both male and female parental caregivers, aged between 18 and 50 years, who were the primary informal caregivers of children with special needs aged 18 years or younger and who had provided informed consent. Special needs children were defined as those diagnosed with conditions including attention deficit hyperactivity disorder (ADHD), autism spectrum disorder (ASD), cerebral palsy (CP), Down syndrome, CP with Down syndrome, or intellectual disability (ID). Exclusion criteria were parental caregivers who had undergone surgery, pregnant or lactating women, fathers with a history of alcoholism, and caregivers diagnosed with psychotic disorders.

### Questionnaires Used in the Study

The study focused on specific psychological variables involving the administration of four questionnaires: The Zarit Burden Interview–Caregiver Burden Scale,^5^ Depression Anxiety Stress Scale,^6^ Pittsburgh Sleep Quality Index,^7^ and World Health Organization Quality of Life–brief form.^8^ The questionnaires were translated into Tamil by the first author (P.F.P.) and corresponding author (A.B.B.) for the purpose of this research, using the standard forward–backward translation method, as recommended by the World Health Organization for cross-cultural adaptation of research tools.^9^

The questionnaires were selected based on their validated application in parental caregiver research and their ability to offer a comprehensive evaluation of the mental health of caregivers of special needs children. Combining these tools ensured a multidimensional assessment of parental caregiver well-being, incorporating both subjective and objective measures that have been supported by previous research.

#### Zarit Burden Interview–Caregiver Burden Scale

Caregiver burden was assessed using the standardized 22-item Zarit Burden Interview–Caregiver Burden Scale (ZBI-CBS) developed by Zarit et al.^5^ This tool aims to capture the emotional impact of caregiving, recognizing the stress associated with this role. The first 21 questions offer five categorical responses (Never, Rarely, Sometimes, Quite Frequently, and Nearly Always), rated 0 to 4, respectively. Question 22 introduces a different set of responses (Not at All, A Little, Moderately, Quite a Bit, and Extremely), also rated 0 to 4, respectively. Scores are derived by summing up the ratings of each item, with higher totals indicating a greater burden. The scoring range is 0 to 88. Burden levels are categorized as: Little or No Burden (0–20); Mild to Moderate Burden (21–40); Moderate to Severe Burden (41–60); and Severe Burden (61–88).

#### Depression Anxiety Stress Scale

The Depression Anxiety Stress Scale (DASS-21) is a scale aimed at gauging an individual’s emotional states related to depression, anxiety, and stress.^6^ This self-reporting instrument consists of three scales, Depression, Anxiety, and Stress, totaling 21 items. Each scale comprises seven items, categorized into subscales with similar content.

The Depression scale assesses various aspects such as hopelessness, devaluation of life, dysphoria, self-deprecation, and lack of interest or involvement. It provides insights into the emotional landscape related to depressive states. Scoring ranges from minimum (0–9, normal) to maximum (28+, extremely severe). The Anxiety scale aids in evaluating situational anxiety, skeletal muscle effects, autonomic arousal, and the subjective experiences of anxiety. This scale delves into the multifaceted aspects of anxiety, including both physical and emotional dimensions. Scores range from minimum (0–7, normal) to maximum (20+, extremely severe). The Stress scale contributes to understanding factors like being easily upset, irritability, over-reactivity, impatience, nervous arousal, and difficulty relaxing. It offers insights into the individual’s responses to stressors and their ability to cope with stress. Scores range from minimum (0–7, normal) to maximum (20+, extremely severe). Overall, the DASS-21 provides a comprehensive assessment of an individual’s emotional well-being, specifically focusing on depression, anxiety, and stress through its distinct scales and subscales.

#### Pittsburgh Sleep Quality Index

The Pittsburgh Sleep Quality Index (PSQI) serves as an effective tool for assessing the quality and sleep patterns of adults over the preceding month.^7^ Comprising 19 individual items, organized into 7 components, it ultimately generates a comprehensive global score. The differentiation between “poor” and “good” sleep quality is achieved by evaluating seven specific areas (components): subjective sleep quality, sleep latency, sleep duration, habitual sleep efficiency, sleep disturbances, use of sleeping medications, and daytime dysfunction over the last month. The scoring system ranges from 0 to 21 with scores of 5 or below indicating good sleep quality, and scores above 5 indicating poor sleep quality; a high score would indicate very poor sleep quality.

#### World Health Organization Quality of Life–Brief Form

The World Health Organization Quality of Life–brief form (WHOQOL-BREF) comprises 26 items and was primarily designed to assess quality of life. It evaluates four key domains: physical health, psychological health, social relationships, and environment. Each domain score reflects the individual’s perception of their quality of life (QoL) within that specific aspect. The scoring is oriented positively, with lower scores indicating a lower QoL and higher scores signifying a higher QoL. To calculate the domain score, the mean score of each domain item is calculated. Scores below 50 are indicative of a low QoL, reflecting significant challenges in health, psychological well-being, or social relationships. Xia et al. categorized scores under 50 as reflecting poor QoL in their study of Chinese community residents, consistent with findings in similar populations.^8^ Scores between 50 and 75 are considered indicative of moderate QoL, where individuals are relatively stable but may still face some challenges, as noted in studies by Benítez-Borrego et al. and Skevington et al.^10^,^11^ Finally, scores above 75 suggest a high QoL, reflecting positive health outcomes and overall well-being, as shown by Chan et al.^12^ These score ranges allow for a nuanced interpretation of QoL in different domains, providing a reliable framework for assessing and comparing QoL across diverse populations.

### Study procedure

A data collection form was used to document participant sociodemographic variables. Four questionnaires were administered to collect data on the psychological variables, as detailed above.

Based on India’s threshold poverty line in the year 2023–24, the monthly per capita consumption expenditure (MPCE) in rural and urban areas is estimated to be rupees (INR) 4,122 and INR 6,996, respectively.^13^ The study area was classified as urban, hence income details were framed accordingly.

### Statistical Analysis

Data analysis was performed using Microsoft Excel® software and Statistical Package for Social Sciences (SPSS Statistics for Windows, Version 16).

For descriptive analyses, mean and standard deviation were used to depict the distribution of continuous variables; frequency and percentages were used for depicting data distribution pertaining to the categorical variables.

## RESULTS

### Socio-demographic Profile

A total of 66 parents participated in the study, with a mean±SD age of 38.4±6.6 years. The sociodemographic data are presented in Table 1. Most study participants were females, and more than half were married. Most were unemployed/homemakers; more than half of the participants were unskilled workers. The majority of parents were Hindu, and most were part of a nuclear family (Table 1).

**Table 1:** Sociodemographic Profile of Parental Caregivers (*n*=66).

Variable	Category	Number (*n*, %)
Parental sex	Female	53 (80.3%)
	Male	13 (19.7%)

Marital status	Single	3 (4.5%)
	Married	51 (77.3%)
	Separated	3 (4.5%)
	Widowed	9 (13.6%)

Income per month	Up to INR 15,000	31 (46.9%)
	INR 15,001–25,000	6 (9.1%)
	INR 25,001–50,000	4 (6.1%)
	INR 50,001–100,000	1 (1.5%)
	No response	24 (36.4%)

Employment status	Employed full time	18 (27.3%)
	Employed part time	7 (10.6%)
	Unemployed	25 (37.9%)
	No response	16 (24.2%)

Occupation	Skilled	5 (7.6%)
	Unskilled	35 (53.0%)
	No response	26 (39.4%)

Religion	Christian	4 (6.1%)
	Hindu	57 (86.4%)
	Muslim	4 (6.1%)
	No response	1 (1.5%)

Family structure	Nuclear family	52 (78.8%)
	Joint family	13 (19.7%)
	No response	1 (1.5%)

INR, Indian rupees.

Our study provides evidence that caregiving significantly impacts the quality of sleep for parental caregivers. The majority of subjects experienced poor sleep quality. The range of caregiver burden varied widely among parents, spanning from minimal to severe levels. The majority of the parents experienced depression, anxiety, and stress (Table 2).

**Table 2:** Scales Used in the Study with Scoring Criteria and the Study Scores.

Name of Scale		How Scored	*n* (%)
The Zarit Burden Interview-Caregiver Burden Scale (ZBI-CBS)		No to minimal burden (0–20)	10 (15.2%)
		Mild to moderate burden (21–40)	24 (36.4%)
		Moderate to severe burden (41–60)	28 (42.4%)
		Severe burden (61–88)	4 (6.1%)

The Depression Anxiety Stress Scale (DASS–21)	Depression	Normal (0–9)	7 (10.6%)
		Mild depression (0–13)	23 (34.9%)
		Moderate depression (14–20)	31 (46.9%)
		Severe depression (21–27)	5 (7.6%)

	Anxiety	Normal (0–7)	7 (10.6%)
		Mild anxiety (8–9)	25 (37.9%)
		Moderate anxiety (10–14)	28 (42.4%)
		Severe anxiety (15–19)	6 (9.1%)

	Stress	Normal (0–14)	9 (13.6%)
		Mild stress (15–18)	25 (37.9%)
		Moderate stress (19–25)	32 (48.5%)

The Pittsburgh Sleep Quality Index (PSQI)		Good sleep quality (0–5)	4 (6.1%)
		Poor sleep quality (>5)	62 (93.9%)

The World Health Organization Quality of Life–Brief Form (WHOQOL–BREF), mean value±SD*		Physical domain	39.3±10.1
		Psychological domain	40.38±10.6
		Social domain	42.61±12.9
		Environmental domain	42.64±11.4

* Since the WHOQOL-BREF form has no categorization levels, scores are confined to mean values.

We investigated the quality of life among parents: the WHOQOL-BREF scores are generally interpreted on a scale where higher scores indicate a better quality of life across the different domains, which was recorded in Table 2.

The parental caregivers in the study reported moderate to low quality of life across various domains. In physical health and psychological health, their scores were notably below average. Social relationships and environmental factors were slightly better. Overall, physical and psychological health appear to be particularly challenging for these parental caregivers (Table 2).

## DISCUSSION

The current study was conducted to evaluate the psychological health status of the parental caregivers of children enrolled in a special education school in Puducherry by evaluating their levels of caregiver burden, depression, anxiety, stress, quality of sleep, and quality of life. Our observation was similar to other studies with reference to gender distribution and marital status.

### Levels of Caregiver Burden Among Parental Caregivers

In the present study, a significant proportion of parental caregivers experienced moderate to severe burden. This contrasts with the findings of Alam El-Deen et al., who used the same ZBI-CBS tool among caregivers of children with Down syndrome.^14^ They reported that 51.9% experienced little or no burden, and only 7.4% had moderate to severe burden. Similarly, Albayrak et al. found significantly higher ZBI-CBS scores in caregivers of children with cerebral palsy compared to controls, although burden categories were not reported.^15^ These comparisons highlight the relatively high burden observed in our study population.

### Levels of Depression, Anxiety, and Stress Among Parental Caregivers

This study highlighted significant rates of anxiety and depression among parental caregivers, underscoring the impact of their responsibilities on mental well-being. Reilly et al.^16^ found that 72% of mothers and 49% of fathers of young children with epilepsy scored in the at-risk range on at least one DASS-21 subscale—that is, their scores exceeded published cutoffs for mild to severe symptoms of depression, anxiety, or stress. Lim et al.^17^ evaluated DASS-21 scores in Singaporean parents of special needs children during the COVID-19 pandemic. Notably, their DASS-21 scores were inversely correlated with resilience, highlighting the protective role of resilience in this population. These findings are consistent with our results, in which the majority of caregivers had scores suggesting levels of depression, anxiety, and stress beyond the normal range on all three DASS-21 subscales, emphasizing the psychological toll of caregiving in this population.

Parental stress is known to negatively influence parenting behaviors and child outcomes, even in families without disability. For example, Păsărelu et al.^18^ found that higher parental stress—measured in parents of neurotypical children—was associated with negative parenting practices and increased emotional and behavioral difficulties in children. While their study did not include special needs populations, it reinforces the broader impact of caregiver distress on child well-being. Given the elevated DASS-21 scores observed in our study, these effects may be even more pronounced in caregivers of children with special needs.

### Quality of Sleep of Parental Caregivers

The findings indicated that most parental caregivers experienced poor sleep quality, underscoring the significant influence of caregiving responsibilities on sleep patterns. Similar results were reported by Albayrak et al.,^15^ who found that mothers of children with cerebral palsy had significantly poorer sleep than did control participants. Pasin et al.^19^ likewise reported poor sleep quality as a key symptom in caregivers with chronic fatigue syndrome, and Ergenekon et al.^20^ found that sleep disturbances among caregivers of children with spinal muscular atrophy were closely linked to depression and anxiety symptoms.

### Quality of Life Among Parental Caregivers

In the present study, the scores obtained from the WHOQOL-BREF questionnaire were interpreted using established and previously validated score ranges.^8^–^12^ Previous studies have reported decreased QoL among parental caregivers of younger children, children with multiple health issues, or children displaying poor social adaptation.^21^–^23^ Specifically, Pokharel et al. studied caregivers of children with epilepsy, demonstrating notably lower QoL scores associated with poor seizure control and economic hardship.^21^ Although these findings align with our observations, generalizing these results to broader special needs populations should be done with caution. Additionally, Patel et al. investigated caregivers of children with autism spectrum disorder (ASD), revealing significant caregiver burden and compromised QoL, particularly influenced by socioeconomic factors and symptom severity.^22^ This context-specificity should be clearly recognized when interpreting comparative findings.

The present study included parental caregivers of children with diverse special needs conditions, specifically attention deficit hyperactivity disorder (ADHD), autism spectrum disorder (ASD), cerebral palsy (CP), Down syndrome, CP with Down syndrome, or intellectual disability (ID). Although this did not encompass every possible special needs condition, our multidimensional assessment—incorporating caregiver burden (ZBI-CBS), psychological well-being (DASS-21), sleep quality (PSQI), and overall quality of life (WHOQOL-BREF)—significantly mitigated condition-specific biases. Future research should continue to examine caregiver experiences specific to individual diagnoses, but our findings clearly underscore a universally high burden experienced by parental caregivers, regardless of their child’s specific condition.

Considering the consistency of findings between our study and others, effective early interventions aimed at reducing caregiver burden and improving their quality of life should be prioritized. Several studies have demonstrated that practices such as yoga,^23^–^25^ Indian aesthetic dance,^25^ mindfulness-based stress reduction (MBSR),^26^ and self-compassion programs^27^ can significantly alleviate mental health challenges among parental caregivers of children with special needs. Additionally, respite programs^28^ and nutrition classes^24^ have been recognized as practical interventions for stress reduction and enhancing overall well-being. Although outside the scope of this study, these interventions represent promising approaches for mitigating the caregiving-related stress.

## STRENGTHS AND LIMITATIONS

There is a paucity of research examining caregiver burden, stress, depression, anxiety, quality of sleep, and quality of life in parental caregivers in Puducherry, India. This study provides valuable insight into the mental impacts on parental caregivers and highlights the need for regular psychological monitoring and targeted interventions for this group.

This study has several limitations. It was a single-center study, and the majority of participants were mothers. Data on emotional and behavioral functioning of the children were not collected; this precludes correlation analyses with the caregivers’ mental health. The lack of a control group consisting of parental caregivers of healthy children prevented a more significant comparison between the two groups. Although we did not specifically compare the demand on caregivers across different special needs conditions, our comprehensive approach of evaluating multiple domains had partly overcome this limitation, thereby improving the overall generalizability of our findings.

## CONCLUSION

Our study highlights the prevalence of depression, anxiety, stress symptoms, poor quality of sleep, caregiver burden, and reduced quality of life encountered among parental caregivers in Puducherry. These findings are consistent with previous studies and deepen our understanding of the impact of challenges faced by parental caregivers of special needs children.

By using four complementary assessment tools, our data provided a more comprehensive view of caregiver burden than would have been possible with any single instrument alone. Given the substantial health burden identified herein, future research should focus on evaluating the effectiveness of alternative interventions by applying these multidimensional measures both before and after intervention.

Overall, our results underscore the critical need for targeted support and intervention programs to alleviate the impact of caregiving on parents, thereby improving their mental health and overall well-being.

## Abbreviations

CRIN: Child Rights Information Network
DASS-21: Depression Anxiety Stress Scale
PSQI: Pittsburgh Sleep Quality Index
WHOQOL-BREF: World Health Organization Quality of Life–brief form
